# Long-term observation of *Magnetospirillum gryphiswaldense* in a microfluidic channel

**DOI:** 10.1007/s00203-019-01713-0

**Published:** 2019-08-14

**Authors:** Tijmen A. G. Hageman, Marc P. Pichel, Per A. Löthman, Jiung Cho, Miri Choi, Nuriye Korkmaz, Andreas Manz, Leon Abelmann

**Affiliations:** 1 KIST Europe, Saarbrücken, Germany; 2 University of Twente, Enschede, The Netherlands; 3 Saarland University, Saarbrücken, Germany; 4 KBSI, Seoul, Korea

**Keywords:** Magneto-tactic, Bacteria, Velocity, Microfluidic

## Abstract

We controlled and observed individual magneto-tactic bacteria (*Magnetospirillum gryphiswaldense*) inside a $5\, \upmu \hbox {m}$-high microfluidic channel for over 4 h. After a period of constant velocity, the duration of which varied between bacteria, all observed bacteria showed a gradual decrease in their velocity of about $25\, \hbox {nm}/\hbox {s}^2$. After coming to a full stop, different behaviour was observed, ranging from rotation around the centre of mass synchronous with the direction of the external magnetic field, to being completely immobile. Our results suggest that the influence of the high-intensity illumination and the presence of the channel walls are important parameters to consider when performing observations of such long duration.

**Electronic supplementary material:**

The online version of this article (10.1007/s00203-019-01713-0) contains supplementary material, which is available to authorized users.

## Introduction

Long-term microscopic observation can help us to understand bacteria behaviour, such as cell division (Inoue et al. 2001) or transition into a growth-arrest phase (Gefen et al. 2014). These experiments are most easily performed on non-motile bacteria. Long-term observation of motile bacteria is far more difficult, but interesting. Do they always swim, also during cell division?

When we want to observe motile rather than fixed bacteria in an optical microscope, there are two additional issues: (1) the bacteria move out of the field of focus and (2) they move out of the field of view. Microtechnology offers a solution to the first problem, since it allows us to fabricate microfluidic channels with a channel height of a few $\upmu \hbox {m}$, so that the bacteria can be forced to remain in focus (within the depth of field). Magneto-tactic bacteria (MTB) (Klumpp et al. 2018) offer a solution to the second problem, since they can be forced to swim along a predefined pattern by an external magnetic field sequence in feed-forward (Pichel et al. 2018) or even feed-back (Khalil et al. 2013).

In this paper, we describe an experiment in which we observed individual MTB swimming in a figure-8 pattern for a duration of several hours. Our question was how the velocity of the bacteria develops over time, and what happens if the MTB stop swimming. This question has relevance for the application of MTB as carriers for targeted drug delivery (Felfoul et al. 2016), in which the MTB will be travelling for 30 min or more towards the tumor site. The genes for magnetosome formation are being identified (Uebe et al. 2018), and it is being investigated whether they can be expressed in other types of microorganisms (Kolinko et al. 2014). Novel venues can be taken for cells acting as drug delivery agents. If cells from the human microbiota can be genetically engineered to express magnetosomes, their lifetime in the human body is likely to be larger than that of cells not naturally occurring in the human body. Also the type and number of tasks that such magnetically steerable cells can perform may increase. Long-term behaviour is also of importance for the application of MTB as transporters inside microfluidic systems themselves (Chen et al. 2014).

As far as we know, there have been no observations of individual swimming bacteria over a period of more than a few minutes. MTB have been observed in chambers fabricated from cover slides glued to microscope slides. Reufer et al. (2014) observed the trajectories of MSR-1 MTB swimming along the top or bottom surface for a few seconds. Erglis et al. (2007) used $28\, \upmu \hbox {m}$-thick double-sided tape to reduce the cell height, and observed single MSR-1 for a period up to 200 s.

There are no reports on long-time observations of individual MTB in microfabricated chips. Other motile bacteria have been observed (*E. Coli* in a channel with a height of $60\, \upmu \hbox {m}$ (Ahmed and Stocker 2008) and *S. marcescens* in a channel with a height of $10\, \upmu \hbox {m}$ (Binz et al. 2010)), but observation times have been below 1 min. Männik et al. (2009) observed the growth of a culture of *E. Coli* up to 2 days in microfluidic chips with channel heights of 5–$7\, \upmu \hbox {m}$, but did not track single bacteria for longer than a few seconds.

When one observes motile bacteria inside microfluidic channels, the analysis of motion is complicated by the presence of the channel walls. In general, moving bacteria are attracted to walls because their flow field is mirror symmetric with respect to the plane of the wall. In combination with random motion, this leads to a preferred distance of travel at several tens of $\upmu \hbox {m}$ from the surface (Berke et al. 2008). In shallow channels, near-surface effects start to play a role, for instance because the flagella interacts with the channel wall. When the distance between the flagella and the wall drops below a few tens of nm this leads to rotating bacteria trajectories (Lauga et al. 2006).

To avoid wall effects, one can use larger volume containers that are moved mechanically in a tracking microscope. To keep the bacteria in focus, the tracking has to be done in three dimensions (Taute et al. 2015). The distance between the bacterium and the top of the container is limited by the working distance of the lens, at most a few mm. The longest trajectories observed therefore are in the order of 100 s. This method has not been applied for magneto-tactic bacteria, which would be an interesting approach, especially if closed-loop feedback is used to keep the bacterium in a virtually confined volume.

In this work, we observed MSR-1 inside a glass microfabricated microfluidic chip with a channel height of only $5\, \upmu \hbox {m}$, to ensure they stay in the depth of field during the entire experiment. In combination with magnetic control, we could observe individual MTB swimming in a figure-8 pattern. The bacteria were observed one by one, for periods up to 70  min, for a total duration of the experiment of 260  min. During the experiment, we monitored magneto-tactic bacteria travelling as far as 14 cm, which is much longer than any of the experiments reported so far.

## Experimental

### Cultivation of the magneto-tactic bacteria

*Magnetospirillum gryphiswaldense* strain MSR-1 (DSM 6361) cells were grown heterotrophically in a liquid medium (pH 7.0) containing succinate as the energy source, as previously described by Lefèvre et al. (2014). $\hbox {FeSO}_4$ was used as the iron source. The MSR-1 cells were cultivated at $26\,^{\circ }\hbox {C}$ for 3–5 days in sterile microfuge tubes prior to microscopic analyses.

**Fig. 1:**
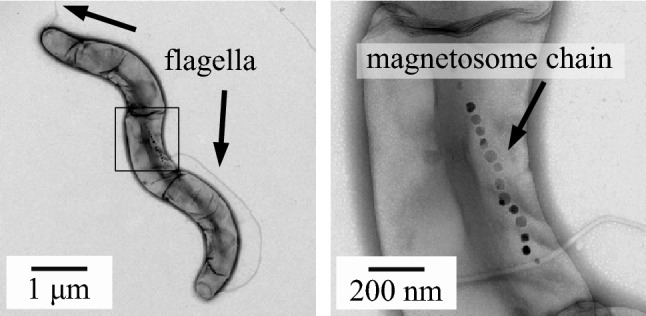
Bright field TEM images of the MRS-1 magneto-tactic bacteria. In this negatively stained image, the flagella can be clearly observed (left) as well as the magnetosome chain (right)

The sampling was done using a magnetic “racetrack” separation method as described in Wolfe et al. (1987). Figure 1 shows a transmission electron microscope (TEM) image of an MSR-1, in which the magnetosome chain can be clearly identified.

### Transmission electron microscopy imaging

For the TEM analysis, the medium with MSR-1 MTB was applied on carbon-coated copper grids (CF200-Cu) and allowed to absorb for 30 s. Excess sample was blotted off by touching the edge of the grid with a clean piece of filter paper and stained with 2% uranyl acetate solution for 30 s. The morphology of the MTB was examined by a JEM-2100F TEM (JEOL, Japan) with bright field image at an accelerating voltage of 200 kV.

### Microscope

**Fig. 2:**
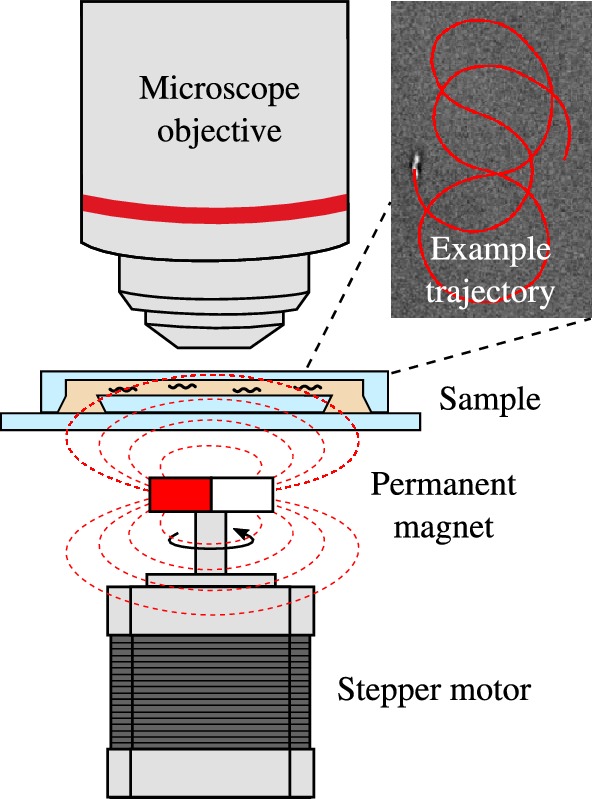
A sample of MTB is inserted in a sealed microfluidic chip and observed with a reflected light microscope. A motorized magnet located under the sample generates in-plane magnetic fields, used to keep the bacteria in the field of view

Figure 2 shows the experimental setup. We used an upright reflected light microscope (Zeiss Axiotron II) with a $20\times$ lens with a numerical aperture of 0.5 optimized for reflected light, a working distance of 2.1 mm, and field of view of 25 mm (Zeiss Epiplan HD DIC). Images were taken by a CCD camera (Point Grey FL3-U3-13S2M-CS) at 10 fps with a resolution of $1328 \times 1024$.

As light source, a collimated blue LED with an average wavelength of 470 nm was used (Thorlabs M470L2-C4). The manufacturer specifies an approximate beam power of 210 mW in a beam diameter of 37 mm. Upon $20 \times$ magnification, this leads to a theoretical power density of $78~{\hbox {kW/m}}^2$. Since for imaging the aperture is nearly closed, a large fraction of the power is blocked. By measuring the intensity difference between a fully open and nearly closed aperture by means of the average intensity on the camera, we estimate a reduction by a factor of 30, leading to an estimated power density of approximately $3~{\hbox {kW/m}}^2$.

### Microfluidic chip

**Fig. 3:**
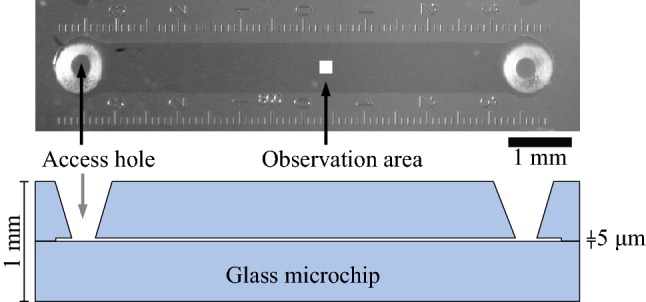
A microfluidic channel with a height of only $5\, \upmu \hbox {m}$ was used for the observation. MTB can be loaded through the powder blasted access holes. The field of view was $200\, \upmu \hbox {m}$ (white box). MTB were redirected before drifting out of the centre of the field of view

The MTB were observed inside a microfluidic chip with a channel height of $5\, \upmu \hbox {m}$, identical to our experiments in Pichel et al. (2018). An overview of the microfluidic chip and region of interest for observation can be seen in Fig. 3. The microfluidic chip was positioned with its entry holes facing a microscope slide, using a thin layer of vaseline between the chip and the seal to reduce evaporation of the liquid from the channel. This sealing method is sufficient for several hours of observation.

### Magnetic field

**Fig. 4:**
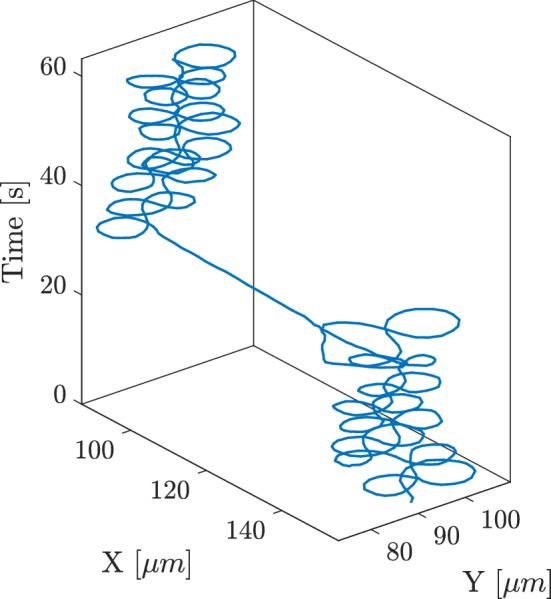
Re-centring maneuver to keep a given MTB in the field of view. The long straight path indicates a manual correction between sequences of figure-8 loops

The magnetic field is generated by a motorised permanent magnet placed underneath the sample (Pichel et al. 2018). This magnet has its magnetisation orthogonal to the axis of rotation, so that it creates an in-plane magnetic field at the location of the sample. The in-plane angle of the field can be controlled by the rotation of the motor axis. The motor was programmed to loop in a figure-8 trajectory, so that the bacteria, on average, will not change their position. The programmed trajectory can be manually overridden, to steer the bacteria back to the centre of view to correct for drift. The tracking results of a typical re-centering process can be seen in Fig. 4. The total duration of the experiment was 5 h. The operators took turn in monitoring the bacterium, and correcting for drift. Towards the end of the experiment, the liquid in the input channels evaporated and the experiment had to be terminated.

### Image processing

The image sequence was processed offline to extract the coordinates of the bacteria of interest. The procedure is identical to the method published in Pichel et al. (2018). The low-contrast nature of the image required pre-processing steps. Subsequently, we performed background subtraction, lowpass filtering, thresholding, and finally selecting the resulting blobs based on size. The centre of gravity was registered as the position of the bacteria. A nearest-neighbour algorithm with maximum search radius was used to build the trajectories from the detected bacteria. The resulting trajectories were manually cleaned for drop-outs. The velocity was calculated from the trajectories. Due to noise, the centre of gravity of the blobs jitters by one or two pixels (180 nm per pixel). As the velocity is calculated from the frame-by-frame displacement at 10 fps, this will result in a small residual velocity of approximately $3 \, \upmu \hbox {m}/\hbox {s}$.

## Results

### Long-term tracking

Data were recorded for a period of 5 h. During this period, one MTB was tracked at a time. An example of a recorded video is available as additional material (figure8.mov). When the selected MTB stopped moving, the magnetic field was directed to be parallel to the microfluidic channel in order to find and trap a new MTB. Over the duration of the experiment, five individual bacteria could be observed.

**Fig. 5:**
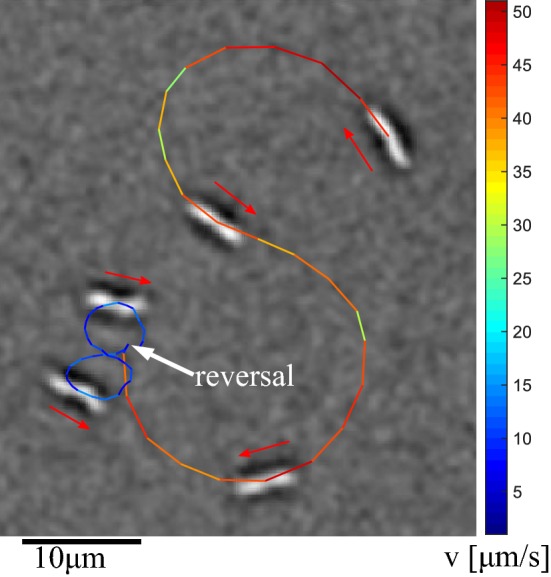
Two figure-8 trajectories of a single MTB. At the start of the observation, the MTB travels at a velocity of 30–$50 \, \upmu \hbox {m}/\hbox {s}$ resulting in a big trajectory. After $2.4 \, \hbox {s}$ the MTB reverses direction and its velocity drops to $5 \, \upmu \hbox {m}/\hbox {s}$, resulting in a much smaller trajectory

Figure 5 shows a composite image of one MTB. The total trajectory with a length of 6 s is shown. On top of the trajectory five frames of the recorded movie are superimposed. At the start of the observation, the MTB follows big figure-8 shaped trajectories at a velocity of up to $50 \,\upmu \hbox {m}/\hbox {s}$. After $2.4~\hbox {s}$, the MTB reverses direction and continues to swim at a very low speed of $5 \, \upmu \hbox {m}/\hbox {s}$, resulting in a small figure-8 shaped trajectory. This behaviour is in agreement with the bimodal velocity distribution previously observed by Reufer et al. (2014).

**Fig. 6:**
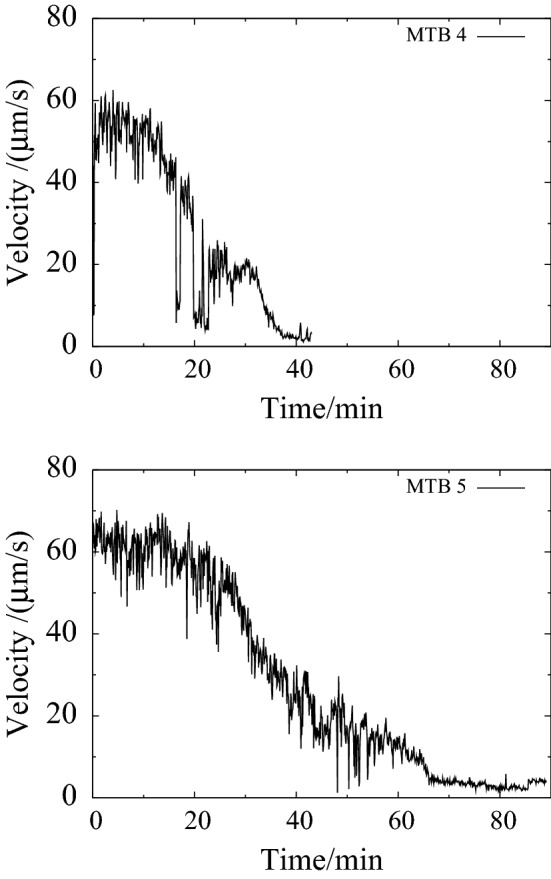
Velocity of two individual MTB versus time. The initial velocity is 50–$60 \, \upmu \hbox {m}/\hbox {s}$. The velocity decreases with time over a period of about half an hour, until the MTB stop moving

Figure 6 shows the velocity of two MTB as functions of time. The MTB initially show a constant velocity, after which the velocity gradually decreases with time. The initial velocity and the duration of the period of constant velocity vary. For MTB4, which is the same MTB as in Fig. 5, several reversals can be observed between 18 and 22 min.

**Fig. 7:**
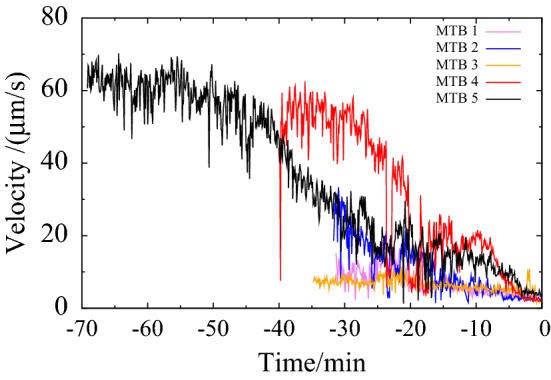
Velocity of observed MTB as a function of time. The time at which the bacteria stops moving is taken as reference ($t\,=\,0$). This way of displaying clearly suggests that the decrease in speed shows a similar behaviour between the MTB

There appears to be more or less a similar rate, about $1.5 \, \upmu \hbox {m}/\hbox {s}$ per minute ($25 \, {\hbox {nm/s}}^2$), in the decrease of velocity. Therefore we plotted the velocity of all observed MTB as a function of time, taking the point at which the bacteria stops moving forward as a reference, see Fig. 7. The figure suggests that we captured the full behaviour of MTB4 and MTB5, but observed the other MTB at the end of the decay process. This is most likely because we became more skilled in capturing new MTB over the course of the 5 h experiment.

### Behaviour at end of the motile phase

**Fig. 8:**
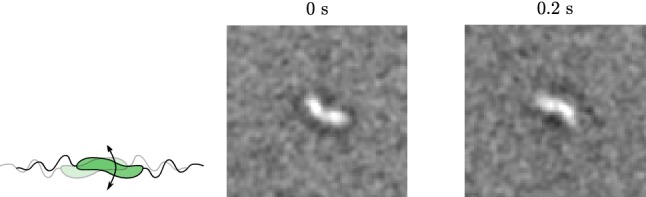
An image sequence of an MTB rotating around the long axis

When the MTB slow down, we can observe a rotation around the long axis of the MTB (Fig. 8), which is in agreement with propulsion by a rotating flagellum (Purcell 1977).

**Fig. 9:**
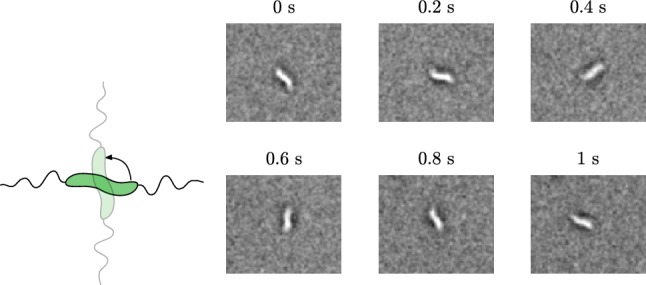
Even if an MTB no longer moves forward, it still is rotating due to the torque generated by the rotating external field. As a result, even non-motile MTB rotate around an axis perpendicular to their body

When the MTB stop swimming, we can still observe movement. The external rotating field will always exert a torque on an MTB with a magnetosome, even if it is dead. We observe these MTB rotating around an axis that appears to be very close to the centre of their body (Fig. 9).

**Fig. 10:**
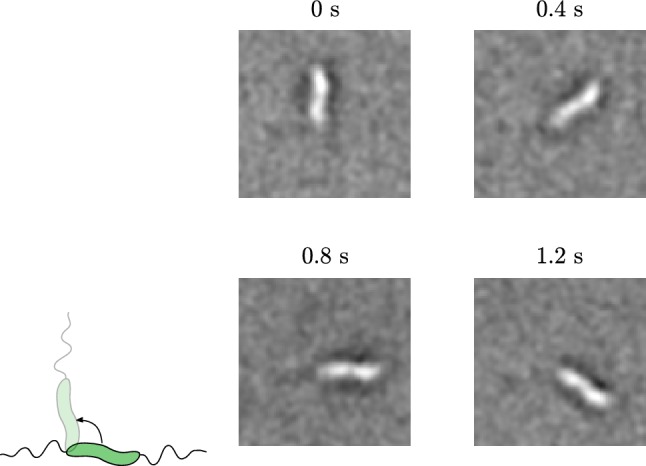
MTB sometimes rotate around one end. These are probably stuck to the channel wall with a flagellum

There are other MTB that appear to be stuck with a flagella to the surface of the channel wall. They rotate around a point that is not in the centre of their body (Fig. 10), remaining aligned with the magnetic field. Also non-magnetic bacteria show this behaviour, but then rotate randomly.

## Discussion

In each case of a long-term control sequence we observe a decline in velocity. There a several hypotheses.

A possible explanation might be that the direct lighting from our observation is heating the samples we are observing to a temperature that kills the MTB. However, no noticeable increase in chip temperature was observed. Since the thermal conductivity of glass is high, it does not support a steep thermal gradient, so the entire chip would be at the same temperature. We were still able to find new motile MTB during a 5 h experiment. It is therefore unlikely that the observation area increases significantly in temperature.

We assume that local ion depletion is also not a problem, since in some cases when an MTB comes to a halt, other non-magnetic ones still cross the screen without a problem.

One could imagine MTB stop swimming prior to cell division. Clear size changes, however, are not visible, even after observing the bacteria for a long period after they stop. From growth curves, we can estimate the mean time between cell division to be on the order of 4–8 h (Sun et al. 2008). The chance that all observed MTB stopped swimming because there were close to cell division is negligible (less than 0.1%).

One might suggest the reduction in velocity is simply due to fatigue of some sort. Perhaps the flagellar motor has intervals of activity over such a long period of time. The efficiency of the flagellar motors is reported to be near 100% (Kinosita et al. 2000). Moreover, we have never observed an MTB starting to move again after coming to a halt.

The most likely explanation is an overdose of light. In the microscope, we focus a very bright LED light source of 450 nm wavelength on the field of view. This high-intensity light source might damage the bacteria under observation. It is known that magneto-tactic bacteria respond to light. For MC-1 bacteria, illumination seems to have the same effect as an increase in oxygen concentration (Frankel et al. 1997). Non-magnetic AMB-1 bacteria have been observed to migrate towards the light (Li et al. 2017). There are no published reports on photo-toxicity of magneto-tactic bacteria, but we could assume a similar sensitivity to light as other types of bacteria. Light illumination at 415 nm at a dose of $750 \, {\hbox {kJ/m}}^2$ for instance strongly reduces the viability of *Propionibacterium acnes* cultures (Ashkenazi et al. 2003). Similarly, Santos et al. (2013) showed that a UV-light dose of $300 \, {\hbox {kJ/m}}^2$ at 365 nm wavelength is sufficient to reduce the viability of most of a set of nine different types of surface water bacteria. Assuming an illumination power density at the sample plane of $3 \, {\hbox {kW/m}}^2$ (see “Experimental” in section), this would be equivalent to an exposure time of less than 250 s.

This photo-toxic explanation is also in agreement with the observation that we can always find a new motile MTB. Only the MTB that are within the illuminated area (about $1.2~{\hbox {mm}}^2$ will suffer from the intense light source. The MTB outside this area remain unaffected until we use them for observation. It is therefore reasonable to assume that indeed an overdose of light is responsible for the decrease in MTB motility over time.

## Conclusion

We observed magneto-tactic bacteria of type MSR-1 inside a microfluidic chip for a total of 260 min. During this time, individual bacteria were magnetically steered in figure-8 patterns for a duration of from 20 to 50 min.

The MTB occasionally reverse direction, which is accompanied with a sudden drop in velocity. All observed bacteria showed a gradual decrease in velocity until they came to a full stop. The time until the start of the decrease varied, with a maximum of 30 min. The decay rate, however, was relatively constant, at about $25~ {\hbox {nm/s}}^2$.

When the MTB slow down but are still swimming, we can observe rotation around their long axis. After coming to a halt, we observed three different behaviors. (1) Many MTB still rotate in the field around an axis perpendicular to their long axis and close to their centre of mass. (2) Some MTB appear to be stuck to the channel wall with a flagellum and rotate around one end. They either rotate synchronously with the rotation of the field or randomly. The latter group must be non-magnetic. (3) Finally there are MTB that do not move at all, either because they are firmly stuck or non-magnetic.

As far as we know, this experiment is the first observation of individual motile bacteria for an extended period of time. The experiment was enabled by the availability of glass microfabricated chips with low channel height, so that the bacteria stay in focus. We learned from the experiment that one should consider the influence of the microscope light and the presence of the channel walls.

## Electronic supplementary material

Below is the link to the electronic supplementary material.

Supplementary material 1 (mov 1969 KB)
